# Relationships Between Frequency of Attendance, Engagement and Independence in Everyday Activities Among Children and Youth With Intellectual and Other Developmental Disabilities: The Association With Comprehension Difficulties

**DOI:** 10.1111/jar.70209

**Published:** 2026-03-13

**Authors:** Anna Karin Axelsson, Magnus Ivarsson, Anna Ullenhag

**Affiliations:** ^1^ CHILD Research Group, School of Education and Communication Jönköping University Jönköping Sweden; ^2^ Department of Behavioural Sciences and Learning Linköping University Linköping Sweden; ^3^ Academy of Health, Care and Welfare Mälardalens University Västerås Sweden; ^4^ Beitostølen Healthsports Center Beitostølen Norway

**Keywords:** children and youths, comprehension difficulties, independence, intellectual disabilities, participation

## Abstract

**Background:**

Participation (attendance and engagement) is important for children's development and well‐being. The aim was to study the relationships between attendance, engagement and independence in everyday activities of children with intellectual and other developmental disabilities and to compare patterns between children with and without comprehension difficulties.

**Method:**

Participation and independence (FUNDES‐Child‐SE) were measured in 131 Swedish participants (aged 5–18 years). The analysis included Mann–Whitney *U* and Spearman's rank correlation tests.

**Results:**

The group without comprehension difficulties attended with a frequency more similar to typically developing peers, with higher engagement and independence. There were very strong overall attendance‐engagement correlations (*r =* 0.91 for the group with comprehension difficulties and *r =* 0.87 for the group without) and attendance‐independence correlations (*r =* 0.86 and *r* = 0.87), as well as a strong correlation between engagement and independence (*r =* 0.78 and *r* = 0.77).

**Conclusions:**

Despite strong overall correlations between dimensions, differences across specific activity items underscore the need for targeted assessment and intervention.

## Lay Summary

- Children with comprehension difficulties attend everyday activities less often and are less engaged and independent when doing so compared to children with other disabilities.
- There is a strong association between overall attendance, engagement and independence in everyday activities in children with disabilities, but the strength of the association differs across specific activities.
- There is a need for targeted, individualised assessment and intervention in children with disabilities.

## 1 Introduction

Participation in everyday activities, such as dressing, playing games and school activities, is known to be of importance for children's development and well‐being (Ullenhag et al. 2024). According to the International Classification of Functioning, Disability and Health (ICF), participation is defined as ‘involvement in a life situation’ (World Health Organization 2001). This definition has been elaborated on in the Family of Participation‐Related Constructs (fPRC) framework by Imms et al. (2017). In line with previous studies (Granlund et al. 2012; Maxwell et al. 2012), participation in the fPRC is considered to imply both attendance and involvement in activities. Further, attendance is described to be a prerequisite for participation, and involvement, in turn, includes elements of for example, engagement, motivation, persistence, social connection and affect. In tandem with this, the importance of other personal factors is stressed, such as activity competence and preferences. Moreover, independence in activities is closely related to an individual's activity competence and is seen as a facilitating factor for participation. Furthermore, independence is a component closely related to the intrinsic factors of the fPRC model (Imms et al. 2017), such as activity competence and self‐efficacy. When individuals possess the ability to perform an activity, it strengthens their belief in their own capabilities and creates the conditions for feeling involved. This sense of involvement, in turn, increases the likelihood that they will want to participate in similar activities (Nyquist et al. 2020). To encompass a bio‐psycho‐social approach (Engel 1977), the framework also includes environmental aspects, where the context includes other people, places, objects and activities, in which participation is set. The context in the fPRC also includes the aspect of time, meaning that transactional processes (Sameroff 2009) can be considered, that is, how children and the context shape or mould each other over time. Such reciprocal interaction between personal and environmental factors influences the child's participation, both in terms of attendance and involvement, as well as their level of independence.

Previous research studying factors influencing the frequency of attendance, engagement and independence has identified child comprehension difficulties as a significant predictor of all three outcomes (Axelsson et al. 2024). According to the American Association on Intellectual and Developmental Disabilities (AAIDD n.d.), comprehension difficulties (or the ability to understand complex ideas), alongside individual abilities such as planning, problem‐solving and learning from experiences, are key aspects of intellectual functioning. Further, AAIDD stresses that the community context, ability to communicate, behavioural factors and so on, also need to be considered in the diagnosis of intellectual disability. Still, little is known about the interrelationship between the frequency of attendance, engagement and independence in different types of activities performed in different contexts, and whether these interrelationships differ between children with and without comprehension difficulties.

### 1.1 Aim

The aim was to study the relationships between frequency of attendance, level of engagement and independence in everyday activities of children with intellectual and other developmental disabilities, focusing on differences between children with and without comprehension difficulties.

The research questions are:

1. Among children with disabilities, do children with and without comprehension difficulties differ in their level of attendance, engagement and independence in everyday activities?
2. Do the associations between frequency of attendance, engagement and independence differ across activity areas and the two groups?

## 2 Methods

This study has an explorative, cross‐sectional design using quantitative questionnaire data and is part of a longitudinal study on participation and mental health, Child‐PMH.

### 2.1 Participants

In Sweden, children with diagnosed congenital or early acquired pervasive disabilities, such as intellectual disability, autism, cerebral palsy and other movement‐related disorders, are entitled to support and other interventions from habilitation services with the ambition of developing abilities or compensating for impairments and promoting independence and participation in social life. One hundred and thirty‐one caregivers of children with one or more disabilities participated in this study. The children were from their 6th year of age to the end of their 18th year of age and registered at a habilitation centre in six different regions in the middle part of Sweden. The participants represented three different age cohorts as well as two groups based on whether they had comprehension difficulties or not (see Table 1). The habilitation centres were selected by convenience sampling. Informed consent for participation and permission for the publication of results had been obtained from the caregivers of the participating children. The recruitment of participants is described by Axelsson et al. (2024).

**TABLE 1:** Caregiver‐rated level of comprehension difficulties according to the 10th question of the (TQSI) of the 131 included children.

Level of comprehension difficulties	Cohort 1, age 5–6 years 51 participants	Cohort 2, age 11–12 years 56 participants	Cohort 3, age 13–18 years 24 participants	Total 131 participants
None^a^	12 (9%)	24 (18%)	7 (5%)	43 (33%)
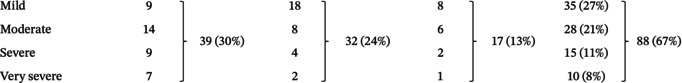				

^a^ The children without comprehension difficulties were also registered at the habilitation services meaning that they had other types of diagnosed disabilities.

### 2.2 Instruments and Variables

This study included a background questionnaire, the participation questionnaire FUNDES‐Child‐SE (Axelsson et al. 2022; Gothilander et al. 2023) and the 10 Questions Screening Instrument identifying children with disabilities (TQSI) (Cappa et al. 2018). In addition to Swedish, the questionnaires were also offered in English, Arabic and Somali. Data was collected from December 2020 to April 2021.

#### 2.2.1 FUNDES‐CHILD‐SE

Data on child frequency of attendance, level of engagement and independence in everyday activities were collected using the caregiver‐rated, validated questionnaire FUNDES‐Child‐SE (Axelsson et al. 2022; Gothilander et al. 2023). The questionnaire originates from the Taiwanese questionnaire Functioning Scale of the Disability Evaluation System (part II)—Child version (FUNDES‐Child) (Hwang et al. 2015). In turn, this Taiwanese questionnaire originates from the Children's Assessment Scale of Participation (CASP), developed to measure participation as one dimension in children with traumatic brain injury. In CASP, the caregiver rates the child's current level of participation across 20 activity areas in comparison to other children of the same age (Bedell 2004). Building on this design, FUNDES‐Child (Hwang et al. 2013, 2015) includes questions about both the frequency of attendance (compared to typically developing peers at the same age) and independence, across the same activity items (Bedell 2004). Items are divided into the same four subsections: (i) ‘Home participation’, (ii) ‘Neighborhood and community participation’, (iii) ‘School participation’ and (iv) ‘Home and community living’. Each activity item is rated on a four‐level response scale (0–3) with lower values denoting more positive outcomes (e.g., higher levels of attendance). The Swedish version of the FUNDES‐Child, FUNDES‐Child‐SE, has thereafter been culturally validated and supplemented with questions about the engagement level in these activity items (Axelsson et al. 2022) in line with the fPRC framework (Imms et al. 2017), as well as with questions about what limits the independence in each included activity item. Consequently, the FUNDES‐Child‐SE measures frequency of attendance, level engagement and independence and factors hindering independence. FUNDES‐Child‐SE has demonstrated acceptable internal consistency and marginal to excellent test–retest ICC (Gothilander et al. 2023). In Sweden so far, a self‐administered version for proxy rating has been used.

#### 2.2.2 Ten Questions Screening Instrument (TQSI)

The TQSI is a reliable, caregiver‐rated questionnaire consisting of 10 binary (Yes/No) items developed to screen for impairments in motor ability, vision, hearing, verbal communication and the ability to be understood and to understand in children in different cultures (Durkin et al. 1995; Cappa et al. 2018). For the present study, the question ‘Compared to other children at the same age, does your child in any way seem to have difficulties to understand or to be slow?’ (nb 10, here interpreted as comprehension difficulties) was supplemented with a follow‐up question for caregivers indicating the presence of impairment: ‘If yes, how would you describe the level of comprehension difficulties?’ On the follow‐up item, a four‐level response scale was used: ‘Mild’, ‘Moderate’, ‘Severe’ or ‘Very severe’.

### 2.3 Statistical Analysis

The differences in mean frequency of attendance, level of engagement and independence for the total summary score, the four subsection summary scores and the 20 included activity item scores of the groups with and without comprehension difficulties were analysed with the Mann–Whitney *U* test, with alpha set at 0.05. A Bonferroni correction was applied to account for the increased risk of Type 1 errors due to multiple comparisons. Due to the ordinal nature of the data, Spearman's rank correlation coefficients were calculated to assess the strength and direction of associations between frequency of attendance, engagement and independence across activities. In interpreting the strength of correlation coefficients, the following thresholds were employed: values between 0.00 and 0.19 were categorised as ‘very weak’, 0.20 and 0.39 as ‘weak’, 0.40 and 0.59 as ‘moderate’, 0.60 and 0.79 as ‘strong’ and 0.80 and 1.0 as ‘very strong’. Correlation coefficients were calculated separately for the groups with and without comprehension difficulties.

## 3 Results

Overall, the group with comprehension difficulties participated in the activities less often, were less engaged and less independent. The correlations between the frequency of attendance, engagement and independence in both groups were strong to very strong; however, there were differences across specific activity items.

### 3.1 Comparisons Between the Groups With and Without Comprehension Difficulties

When comparing the mean total scores between the groups with and without comprehension difficulties, there was a significant difference in all three outcomes (see Table 2). Compared to children without comprehension difficulties, children with comprehension difficulties had significantly lower levels of total frequency of attendance, engagement, and independence in everyday activities. The same pattern was consistent across the four subsections, except for the ‘Home’ and ‘School’ subsections in the engagement dimension, where the differences between the two groups were non‐significant. On the activity item level, the distribution of highly and lowly rated activity items was largely consistent between the dimensions and groups. The highest proportion of significant group differences on specific activity items was observed in the independence dimension (see Table 2).

**TABLE 2:** Means of frequency of attendance (Att), level of engagement (Eng) and independence (Ind), with Mann–Whitney *U* tests for mean differences across disability groups for each FUNDES item, subsection and total score.

	Without comprehension difficulties				With comprehension difficulties				*U*		
	Att	Eng	Ind		Att	Eng	Ind		Att	Eng	Ind
Items											
1. Social, play or leisure activities home with family	0.79	1.07	0.56		1.19	1.55	1.50		2331	2376	2933***
2. Social, play or leisure activities home with friends	1.00	1.00	0.58		1.60	1.65	1.50		2444.5	2479.5	2818***
3. Family chores, responsibilities and decisions at home	0.84	1.28	0.67		1.66	1.88	1.61		2716**	2456.5	2873.5***
4. Taking care of yourself	0.70	1.19	0.91		1.48	1.69	1.69		2721**	2418.5	2668**
5. Moving about in and around the home	**0.19** ^**a**^	**0.42** ^**a**^	**0.16** ^**a**^		**0.53** ^**a**^	**0.72** ^**a**^	**0.61** ^**a**^		2330	2192.5	2374
6. Communicating with other children, youth and adults at home	**0.56** ^**a**^	**0.65** ^**a**^	**0.42** ^**a**^		1.22	**1.14** ^**a**^	**1.28** ^**a**^		2663.5**	2424	2777.5***
7. Social, play or leisure activities with friends outside the home	*1.40* ^b^	1.23	1.02		2.10	2.02	1.88		2602*	2569.5*	2727**
8. Organised activities outside the home	*1.47* ^b^	1.40	1.02		1.90	1.80	1.74		2261.5	2224	2562*
9. Moving around outside the home	0.70	0.74	0.67		1.33	1.56	1.56		2507.5	2667**	2738**
10. Communicate with other children, youth and adults outside the home	0.98	0.91	0.79		1.55	1.49	1.56		2472.5	2464	2596.5*
11. Instructional activities together with classmates	0.93	1.05	1.02		1.45	1.67	1.98		2401.5	2471	2852***
12. Social, play or leisure activities with other students at school	0.95	1.07	0.81		1.45	1.56	1.51		2362.5	2355	2558.5
13. Moving around at school	0.58	**0.72** ^**a**^	**0.49** ^**a**^		**1.01** ^**a**^	**1.23** ^**a**^	**1.19** ^**a**^		2319.5	2400.5	2559.5*
14. Using pedagogical materials and equipment that are also available for other students or that are adapted for you/your child	**0.40** ^**a**^	0.74	**0.49** ^**a**^		**1.14** ^**a**^	1.34	1.42		2570.5*	2426	2708.5**
15. Communicating with other students and adults at the school	0.70	0.77	0.65		1.24	1.30	1.35		2425.5	2420	2571.5*
16. Household activities	0.91	1.19	0.88		1.84	1.99	1.95		2742.5**	2651.5**	2931.5***
17. Shopping and managing money	1.02	0.95	*1.05* ^b^		*2.26* ^b^	*2.30* ^b^	*2.11* ^b^		2957***	2995***	2845***
18. Managing a daily schedule	1.23	*1.42* ^b^	1.23		2.02	2.22	1.90		2628*	2584*	2532.5
19. Using transportation in order to move around in the society	*1.26* ^b^	*1.42* ^b^	*1.02* ^b^		*2.33* ^b^	*2.40* ^b^	*2.30* ^b^		2677**	2580.5**	2925.5***
20. Work and responsibility	1.09	*1.33* ^b^	*1.07* ^b^		*2.22* ^b^	*2.25* ^b^	*1.93* ^b^		2824***	2687**	2646**
Subsections											
Home	4.07	5.60	3.30		7.68	8.61	8.20		2782***	2539.5	3016.5***
Outside the home	4.53	4.28	3.51		6.88	6.86	6.73		2624*	2637.5*	2818***
School	3.56	4.35	3.47		6.30	7.09	7.45		2622*	2556.5	2801***
Home and society	5.51	6.30	5.26		10.67	11.15	10.19		3022.5***	2971.5***	3012.5***
Total scores	17.67	20.53	15.53		31.52	33.72	32.58		2900***	2776**	3018.5***

*Note:* *p < 0.05, **p < 0.01, ***p < 0.001.

^a^ The three activity items with the highest frequency of attendance, level of engagement and level of independence respectively.

^b^ The three activity items with the lowest frequency of attendance, level of engagement and level of independence respectively.

In both groups, there were very strong positive correlations between the overall frequency of attendance and level of engagement, as well as the frequency of attendance and the level of independence. Further, the levels of engagement and independence were strongly correlated. For the subsection scores, the frequency of attendance and level of engagement consistently correlated very strongly in both groups. Regarding correlations between corresponding attendance and independence subsection scores, ‘Outside the home’ scores correlated very strongly in both groups, while ‘Home’ and ‘School’ scores correlated strongly. ‘Home and society’ attendance and independence scores correlated very strongly for the group without comprehension difficulties and strongly for the group with comprehension difficulties. The engagement and independent subsection scores were strongly correlated in both groups except for ‘Home and society’ activities, where they correlated very strongly for the group without comprehension difficulties, and in ‘Home’ activities, where they correlated moderately for the group with comprehension difficulties. At the activity item level, the correlations ranged from weak to very strong in the group without comprehension difficulties, and from moderate to very strong in the group with comprehension difficulties. The items associated with the strongest and weakest correlations differed between the groups. For example, in the group without comprehension difficulties, the items with the weakest correlations between frequency of attendance and engagement were ‘Taking care of yourself’, ‘Moving about in and around the house’ and ‘Using pedagogical materials and equipment …’. In the group with comprehension difficulties, the weakest correlation was observed for ‘Family chores’, ‘Responsibilities and decisions at home’, ‘Social, play or leisure activities with family’ and ‘Communicating with other children, youths, and adults at home’ (see Table 3).

**TABLE 3:** Correlations between frequency of attendance (Att), level of engagement (Eng) and independence (Ind) in everyday activities for each FUNDES item, subsection and total score for participants with and without comprehension difficulties.

	Without comprehension difficulties			With comprehension difficulties		
	Att versus eng	Att versus ind	Eng versus ind	Att versus eng	Att versus ind	Eng versus ind
Items						
1. Social, play or leisure activities home with family	0.78	0.33^b^	0.26^b^	0.70^b^	0.48^b^	0.50^b^
2. Social, play or leisure activities home with friends	0.67	0.52	0.36^b^	0.86	0.52	0.52^b^
3. Family chores, responsibilities and decisions at home	0.75	0.61	0.64	0.67^b^	0.71	0.59
4. Taking care of yourself	0.48^b^	0.78	0.59	0.76	0.82^a^	0.75
5. Moving about in and around the home	0.52^b^	0.50	0.43	0.75	0.90^a^	0.66
6. Communicating with other children, youth and adults at home	0.78	0.44^b^	0.32^b^	0.71^b^	0.71	0.57
7. Social, play or leisure activities with friends outside the home	0.76	0.75	0.59	0.83	0.69	0.53
8. Organised activities outside the home	0.92^a^	0.71	0.67	0.93^a^	0.58	0.54
9. Moving around outside the home	0.65	0.58	0.56	0.80	0.75	0.76^a^
10. Communicate with other children, youth and adults outside the home	0.77	0.71	0.68	0.78	0.83^a^	0.59
11. Instructional activities together with classmates	0.77	0.62	0.62	0.78	0.50^b^	0.63
12. Social, play or leisure activities with other students at school	0.79	0.41^b^	0.38	0.84	0.66	0.60
13. Moving around at school	0.80	0.77	0.78^a^	0.76	0.70	0.76^a^
14. Using pedagogical materials and equipment that are also available for other students or that are adapted for you/your child	0.59^b^	0.71	0.56	0.83	0.81	0.77^a^
15. Communicating with other students and adults at the school	0.87	0.70	0.64	0.77	0.62	0.57
16. Household activities	0.72	0.80^a^	0.74	0.88	0.59	0.57
17. Shopping and managing money	0.81	0.82^a^	0.69	0.92^a^	0.64	0.60
18. Managing a daily schedule	0.85	0.62	0.67	0.82	0.52	0.58
19. Using transportation in order to move around in the society	0.88^a^	0.68	0.75^a^	0.91	0.60	0.53
20. Work and responsibility	0.91^a^	0.85^a^	0.77^a^	0.95^a^	0.50^b^	0.47^b^
Subsections						
Home (item 1–6)	0.85	0.73	0.56	0.80	0.78	0.64
Outside the home (item 7–10)	0.88	0.83	0.74	0.86	0.80	0.65
School (item 11–15)	0.85	0.78	0.74	0.88	0.78	0.76
Home and society (item 16–20)	0.92	0.86	0.87	0.93	0.72	0.64
Total scores	0.91	0.86	0.78	0.87	0.87	0.77

^a^ The three activity items with the strongest correlations in each dimension in the group without and with comprehension difficulties respectively.

^b^ The three activity items with the weakest correlations in each dimension in the group without and with comprehension difficulties respectively.

## 4 Discussion

In this study, the frequency of attendance, engagement and independence in 20 daily activity items was studied in 131 children enrolled in the habilitation services in Sweden. Among these children with different disabilities, the group with comprehension difficulties attended the activities less often, had a lower level of engagement and was less independent than those without comprehension difficulties. In both groups, there were very strong positive correlations of the total scores between the frequency of attendance and the level of engagement, and between the frequency of attendance and the level of independence. The correlation between the levels of engagement and independence was weaker but still strong. On item level, the correlations between specific activity items differed in both groups.

### 4.1 Discussions of Results

The results of this study show that children with disabilities that include comprehension difficulties have lower levels of frequency of attendance, engagement and independence in everyday activities than those without comprehension difficulties. This was evident across the different settings. However, the differences in participation restrictions between the groups were most apparent in the ‘Home and Society’ subscale, on activity items such as ‘Shopping and managing money’, ‘Managing a daily schedule’, ‘Using transportation in order to move around in the society’ and ‘Work and responsibility’. These activities are relatively complex and place higher demands on cognitive abilities, which may help explain the lower frequency of attendance and less engagement in these activities for the group with comprehension difficulties. These results are in line with previous studies demonstrating a lower frequency of attendance (Axelsson and Wilder 2014; Milićević 2022) and level of engagement in activities (Axelsson et al. 2013; Milićević 2022) in children with different types of disabilities. Nevertheless, the results also show that in ‘Home’ and in ‘School’ activities, for example, when it comes to communication and to move around in these environments, children with comprehension difficulties have relatively similar results compared to their peers with other disabilities. This may be due to the fact that these environments are often more structured and adapted to meet the needs of children with comprehension difficulties.

The total scores of the included dimensions were positively and very strongly correlated in all cases, except for engagement and independence, which showed a strong correlation. One possible interpretation of these results is that it might be important to simultaneously support engagement when pursuing independence. Further, as these constructs are somewhat more distinct, they may need to be targeted separately to a higher degree when designing interventions, for example, by making activities fun (Nyquist et al. 2020). Although total scores were strongly correlated, correlations on the item level showed more variability. For example, the correlations between participation (i.e., attendance and engagement) and independence when it comes to ‘Social, play or leisure activities home with family’ were weak for the group without comprehension difficulties and moderate for the group with comprehension difficulties. This pattern could be explained by the fact that families have learned to choose the right occasions for activities, to find suitable activities, or to adjust activities based on the child's interests and abilities. As with participation and independence, relatively weaker correlations were found on the item‐level than on the total score level for attendance and engagement. These results align with findings from an earlier study, which identified attendance and engagement as separate aspects of participation (Gothilander et al. 2024).

This study highlights the importance of creating supportive, up‐to‐date environments to enhance child participation and independence. Such environments should include personal support, assistive devices and technologies and be adapted to the child's needs. Additionally, extra time for learning and achieving goals might be necessary, not least for children with comprehension difficulties. Such aspects are in line with the fPRC model (Imms et al. 2017), which suggests that participation should be considered in relation to the context and environment. Additionally, Sameroff's (2009) transactional theory illuminates how children and the environment shape each other over time. This was also shown by Sjöman et al. (2021), who found that engagement improved over time through positive peer‐to‐child interactions and responsive teachers. Accordingly, there is also a need for persistence in interventions for improved participation and independence.

### 4.2 Discussions of Methods

There is a need to study the aspect of participation in everyday life. The included questionnaire FUNDES‐Child‐SE (Axelsson et al. 2022; Gothilander et al. 2023) includes essential aspects of participation, as well as independence, and additional questions about factors hindering participation in the 20 included activity areas. By adding other aspects of relevance to participation, such as activity competence, preferences and trust (i.e., personal factors) and environmental factors, the questionnaire could yield even richer results. However, such changes would make the questionnaire longer, further increasing the risk of dropouts. The complexity of participation as a multifaceted construct has been highlighted in an interview study by Hammel et al. (2008), which showed that persons with disabilities need the freedom to define and pursue participation on their own terms, with belonging, social connection, choice and control being central aspects. Accordingly, it is possible that deeper knowledge about the participants' participation could have been reached if FUNDES‐Child‐SE had been administered as an interview. At the same time, there is a need for simplification in collecting data about participation by, for example, creating an index or even a classification system, especially for use in comprehensive studies. Interestingly, an example of a shorter participation measure is found in the origin of FUNDES‐Child‐SE, that is, the CASP. In the CASP the respondents are instructed to assess the child's current level of participation only (Bedell 2004), instead of assessing both frequency of attendance and engagement.

This study included three cohorts of children of different ages. The number of participants in each group differed, However, importantly, the frequency‐of‐attendance dimension differs from the independence and engagement dimensions in a key aspect: it instructs respondents to compare the focal child to typically developing children of the same age, making attendance scores less sensitive to age distribution than the other dimensions. Further, Axelsson and Wilder (2014) found that children with profound intellectual and multiple disabilities of different ages overall had a lower level of engagement compared to typically developing peers in everyday activities. Presumably, the greatest impact of a skewed age distribution could be expected in the independence dimension, where older children are likely to be more independent than younger children. Nevertheless, in the study by Axelsson et al. (2024) based on the same sample as the current study, cohort membership was only retained as a weak predictor of independence (and not at all in the attendance and engagement models).

## 5 Limitations

Even if it was possible to differentiate five levels of comprehension difficulties based on the tenth TQSI item, analyses were restricted to a two‐group approach (with/without comprehension difficulties) due to the limited number of participants. By including more participants, it would have been possible to analyse the different levels of comprehension difficulties separately and to provide a more nuanced picture of the variation in participation and independence. Still, the two‐group approach was sufficient to identify comprehension difficulties as an important factor influencing participation and independence patterns. To further strengthen the generalizability of the results, age distribution should have been controlled in a stricter way. Also, the FUNDES‐Child‐SE is a proxy‐report questionnaire; no Swedish version has yet been developed for self‐rating of children. It is recommended that questions about the level of engagement should be self‐reported due to the subjective nature of the phenomenon (Huus et al. 2021). However, the instructions of the FUNDES‐Child‐SE recommended that the questionnaire be answered by a caregiver or another person who knows the child well and, if possible, in collaboration with the child. Further, using proxies may broaden the target group to include also the youngest children and the children with the most cognitive difficulties.

## 6 Conclusions

The presence of comprehension difficulties in children with disabilities is an important factor influencing both participation and independence in everyday activities. Even though strong correlations between the domains of frequency of attendance, engagement and independence in everyday activities were identified, differences in correlation strengths across activity items underscore the importance of assessing each facet separately, especially those of importance for the child. Such specific knowledge about individual needs is necessary when planning appropriate support, over a sustained period, to improve life functioning for children with comprehension difficulties. In this, the (Swedish) habilitation services, after their in‐depth assessment from a bio‐psychosocial perspective, have a considerable responsibility to identify and help implement evidence‐based strategies to remove barriers to participation in the child's different everyday environments. These clinical implications should be tailored to address the specific needs of children, not the least for children with comprehension difficulties. Since these children show a lower frequency of attendance, engagement and independence across various settings, targeted support in these areas could help improve their participation and autonomy. For example, targeted support may include use of a visual schedule, simplified instructions and personal communication tools. Training for caregivers and educators in adapting environments and communication styles may also be beneficial. By focusing on these areas, clinicians can better support the development and integration of children with comprehension difficulties into various environments. To further deepen this knowledge, the open‐ended answers to the question about hindering factors for independence in each of the 20 activities will be analysed in a forthcoming study.

## Funding

This work was supported by the Vetenskapsrådet—Swedish Research Council (grant number 2018‐05824_VR), and Futurum—the Academy for Healthcare, Region Jönköping County (grant number FUTURUM‐989510).

## Ethics Statement

The study has been approved by the Swedish Ethical Review Authority, Dnr 2019‐05028 and Dnr 2017/496‐31. All participants have given their written informed consent to participate.

## Consent

All participants have given their written informed consent to participate.

## Conflicts of Interest

The authors declare no conflicts of interest.

## Data Availability

The data that supports the findings of this study are available on request from the corresponding author.
